# Dramatic response to osimertinib combined with crizotinib in EGFR T790 M mutation only in blood and Met amplification only in tumor tissue expressive non-small cell lung cancer: A case report

**DOI:** 10.1097/MD.0000000000026375

**Published:** 2021-07-30

**Authors:** Dapeng Li, Qi Gui, Caihua Xu, Meng Shen, Kai Chen

**Affiliations:** Department of Oncology, The First Affiliated Hospital of Soochow University, Suzhou, Jiangsu, People's Republic of China.

**Keywords:** EGFR mutation, MET amplification, next-generation sequencing, NSCLC, tyrosine kinase inhibitor

## Abstract

**Rationale:**

Besides the T790 M mutation, it may coexist with bypass pathway activation in real clinical cases for patients with EGFR mutations who resisted to the first- and second-generation tyrosine kinase inhibitors (TKIs) in non-small cell lung cancer (NSCLC). There are limited clinical trial data describing the efficacy of osimertinib combined with MET inhibition in EGFR T790M-mutant NSCLC patients with Met amplification.

**Patient concerns:**

A non-smoking 53-year-old male patient with lung adenocarcinoma underwent gefitinib, afatinib, and osimertinib combined with crizotinib treatment and developed different EGFR resistance mutations.

**Diagnoses:**

The patient was diagnosed with lung adenocarcinoma (stage cT4N2M0, IIIB). After resistance to the therapy targeting EGFR exon 21 L858R point mutation, T790 M mutation was detected in liquid biopsy and Met amplification was detected via tissue biopsy by next-generation sequencing (NGS).

**Interventions:**

The patient received systemic treatments, including chemotherapy, gefitinib, afatinib, and osimertinib combined with crizotinib.

**Outcomes:**

The patient died of multisystem organ failure and had an overall survival of 24 months.

**Lessons:**

Although osimertinib combined with crizotinib therapy showed dramatic tumor shrinkage in both the primary tumor and bone metastasis to an EGFR T790M-mutant NSCLC patient with MET amplification, the progression-free survival (PFS) was only two months.

## 1 Introduction

Advanced non-small cell lung cancer with EGFR gene mutations is closely related to the clinical efficacy of epidermal growth factor receptor tyrosine kinase inhibitors (EGFR TKIs), but the outcomes are limited by acquired resistance.^[1]^ The EGFR exon 20 T790 M mutation accounts for 50% to 60% of the resistance to first- and second-generation TKIs, followed by bypass pathway activation (e.g., MET or ERRB2 amplification, mutations in BRAF and PIK3CA), and lineage shifts (e.g., small-cell transformation, epithelial-to-mesenchymal transition).^[2,3]^ ‘Third-generation’ EGFR TKIs, such as osimertinib, are highly effective in EGFR-mutant NSCLC patients harboring the EGFR T790 M mutation. Nevertheless, resistance to third-generation EGFR TKIs can still develop through further mutations in EGFR and the activation of alternative pathways.^[4]^ With the development of high-throughput NGS-based liquid biopsy technology, additional mechanisms, such as EGFR exon 20 C797S, EGFR L718Q, and EGFR exon 18 G724S mutations were observed.^[4,5]^ NGS-based liquid biopsy testing overcomes spatial heterogeneity and can dynamically monitor tumor genomic evolution and guide decision-making for treatment strategies in a minimally invasive manner.^[6]^

Here, we present the case of an EGFR-mutant advanced NSCLC patient who acquired resistance to first- and second-generation TKIs in a short time. There were different results of the NGS tests between blood samples and tumor tissue samples at the same time, that EGFR T790 M mutation was only found in blood and Met amplification was only found in tumor tissue. Although there are limited clinical trial data describing the efficacy of osimertinib combined with MET inhibition in patients with EGFR T790M-mutant NSCLC, outcomes in a real-world setting are useful to inform clinical practice. Despite disease progression after only 3 months, the patient experienced substantial symptomatic improvement and dramatic tumor regression following osimertinib combined with crizotinib therapy.

## 2 Case presentation

**Figure 1:**
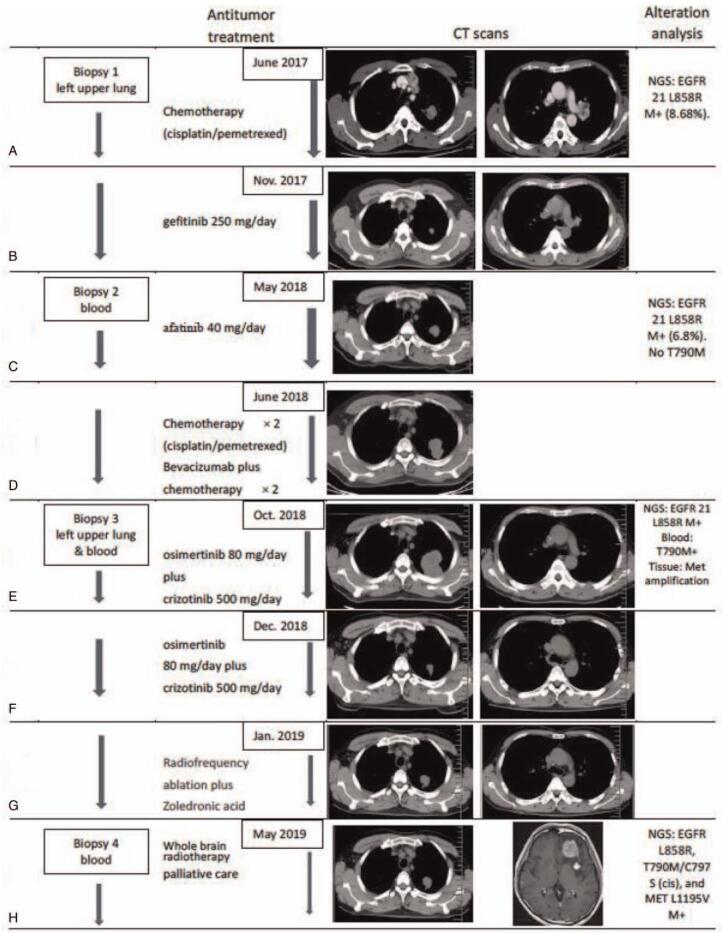
The profile of treatments, images and NGS results of the patient. **(A)** Initial Chemotherapy of cisplatin/pemetrexed had a good response to tumor but caused intolerable adverse reactions. The first NGS suggested EGFR 21 L858R mutation. **(B**) The PFS of first-generation EGFR-TKI gefitinib was only 5 months. **(C**) The second NGS suggested there was no T790 M mutation. And the second-generation EGFR-TKI afatinib was ineffective. **(D**) The PFS of secondary chemotherapy and plus Bevacizumab was only 4 months. **(E**) The third NGS suggested EGFR T790 M mutation was only in blood and Met amplification was only in tumor tissue. Osimertinib combined with crizotinib showed dramatic tumor shrinkage. **(F**) The PFS of osimertinib combined with crizotinib was only 3 months. **(G**) Palliative care for 4 months. **p(H**) Brain metastasis and the fourth NGS of EGFR L858R, T790 M/C797S (cis), and MET L1195 V mutations.

In June 2017, an Asian man aged 53 years presented with productive cough. His past medical history included no history of smoking, minimal exposure to secondhand smoke, and no significant comorbidities. Chest CT revealed a left lung lesion in the upper lobe, with metastases in the left hilar and mediastinal lymph nodes (Fig. 1A), which was confirmed by a subsequent PET scan. Transbronchial biopsy confirmed left lung adenocarcinoma (stage cT4N2M0, IIIB). The biopsy tissue was also sent for NGS testing. However, the NGS process lasted a long time in China at that time, and the report came more than one month later. The patient could not accept that he should wait for a long time without any treatment after being diagnosed with lung cancer. Therefore, he received chemotherapy during the waiting period. He initiated six cycles of cisplatin/pemetrexed chemotherapy (pemetrexed 500 mg/m^2^, day 1 plus cisplatin 75 mg/m^2^, day 1, every 3 weeks) before genotyping results from July 1, 2017 and had a good response on the first evaluation of September 21, 2017. According to RECIST (version 1.1), he reached PR. During this period, NGS testing revealed that the tumor was an EGFR exon 21 L858R point mutation, with an abundance of 8.68%.

Although chemotherapy was effective, the patient declined further chemotherapy because of progressive fatigue and loss of appetite (grade 3 in CTCAE, version 5.0) during chemotherapy. From November 28, 2017 (Fig. 1B), the patient was treated with second-line targeted therapy without disease progression, the first-generation EGFR-TKI gefitinib, at a 250 mg standard daily dose. Poor news was found in April 2018. Computed tomography (CT) showed enlargement of the left upper lobe mass, and the patient experienced disease progression (Fig. 1C).

The tumor in the left lobe was small and isolated in lung tissue, which was behind the back ribs and scapula, so it was difficult to obtain tissue biopsy via either percutaneous lung puncture biopsy or transbronchial biopsy. Therefore, liquid biopsy was conducted, and the results showed an EGFR exon 21 L858R point mutation, but the abundance declined to 6.8% and negative for the T790 M mutation. The patient was treated with second-generation EGFR-TKI afatinib as the third line in May 2018. Only one month later, the mass in the left lobe had progressed (Fig. 1D). Based on the previous effective response to chemotherapy, he received two cycles of cisplatin and pemetrexed (doses were the same as before) during June and July 2018. Chest CT at the end of July 2018 revealed disease stability. Afterwards, bevacizumab, which is a vascular endothelial growth factor inhibitor, was added to combine with cisplatin and pemetrexed chemotherapy for another two cycles from July to August 2018. Unfortunately, a slightly enlarged left lobe mass and left sixth rib bone metastasis were found on chest CT in September 2018 (Fig. 1E). The patient complained of severe chest pain, fatigue, and loss of appetite.

To further identify the potential therapeutic targets, a biopsy was performed on the left lung mass on September 25, 2018 (confirming adenocarcinoma). Immunohistochemistry staining showed high expression of Ki-67 (80%) and PD-L1 (80% of tumor cells). Concurrently, NGS testing of both tumor tissue and blood specimens again identified EGFR exon 21 L858R point mutation, and EGFR T790 M mutation was only found in blood specimens and Met amplification was only found in tumor tissue specimens. Although highly expression of PD-L1, the role of immunotherapy in patients with EGFR-mutated NSCLC remains controversial.^[7]^ And There are several small samples of clinical trial data describing the efficacy of EGFR-TKIs combined with MET inhibition in EGFR-mutant cancers with acquired resistance to Met amplification.^[8,9]^ Therefore, a decision was made to proceed with the third-generation EGFR-TKI osimertinib (80 mg, QD) combined with the MET inhibitor crizotinib (250 mg, BID) from October 17, 2018. The patient still experienced fatigue and loss of appetite during this time, but the symptoms of cough and chest pain were substantially alleviated. CT scans performed in November and December 2018 revealed dramatic tumor reduction and achieved a partial response (Fig. 1F).

This PR response did not last, and the lung tumor mass had progressed in January 2019 (Fig. 1G). After the multidisciplinary committee, the patient underwent radiofrequency ablation of the left lung mass and received zoledronic acid for bone metastasis. Subsequently, there was evidence of new widespread disease in the brain at the end of April 2019 (Fig. 1H), and he also received whole-brain radiotherapy. In May 2019, NGS from blood again showed that EGFR L858R, T790 M/C797S (cis), and MET L1195 V mutations. Currently, there are no targeted therapies against T790 M/C797S available in the clinic, and the patient received palliative care. Eventually, the patient died of multisystem organ failure in June 2019.

## 3 Discussion

The most common acquired resistance mechanism against the first- and second-generation TKIs in EGFR-mutant NSCLC patients might be the T790 M mutation, and other resistant subclones may also coexist in these patients. Amplification of the MET proto-oncogene accounts for 5–20% of acquired resistance to EGFR TKIs.^[9]^ strong expression of c-Met is associated with poor prognosis of NSCLCs.^[10]^ In this case, we analyzed the potential resistance to EGFR mutations during sequential use of the first- and second-generation TKIs (gefitinib and afatinib) in an advanced lung cancer patient. EGFR T790 M was detected in plasma, and MET amplification was found in the tumor at the time of resistance to afatinib. Previous studies have shown that co-occurring genomic events are detected in up to 93% of EGFR-mutant NSCLCs.^[11]^ Tumor biopsies have a greater depth of molecular characterization; however, they may be spatially limited to a minute fragment of patients’ overall cancer burden. The ctDNA comes from multiple disease sites and may illustrate coexistent subclones; thus, liquid biopsies might provide different information about tumor heterogeneity.

MET amplification can drive human epidermal growth factor receptor 3 (ERBB 3)-dependent PI3K activation; therefore, it may be involved in EGFR-TKI resistance.^[12]^ Preclinical studies have demonstrated that in EGFR T790M-mutant NSCLCs, aberrant c-Met activity can confer resistance to third-generation EGFR TKIs.^[13]^ This suggests that inhibition of both c-Met and EGFR T790 M is effective in preventing the growth of EGFR T790M-mutant NSCLCs with c-Met amplification. A recent case report showed that combinatorial treatment with a first/third-generation EGFR-TKI and crizotinib resulted in partial responses in two patients with newly acquired MET amplification after osimertinib resistance.^[14]^ Moreover, in a phase I/II trial of capmatinib (a small-molecule MET receptor TKI) combined with gefitinib in EGFR-mutated tumors after EGFR inhibitor treatment failed NSCLC, the ORR was 47% in patients with MET gene copy number (GCN) ≥ 6 subgroup.^[15]^ In the TATTON study, the objective response rate was 44% in the group treated with osimertinib combined with savolitinib (MET-TKI).^[16]^ Our case confirmed these observations and revealed that EGFR T790 M mutation and MET amplification in different samples can clinically document coexistent, genetically distinct subpopulations differentially responding as predicted by their genotype in real time to therapies. Although the progression-free survival (PFS) of osimertinib in combination with crizotinib was only 3 months, the patient experienced substantial symptomatic improvement in chest pain and achieved partial responses.

Further liquid biopsy provided insight into clonal dynamics after treatment failure with osimertinib combined with crizotinib, emergence T790 M/C797S. A previous study reported that the EGFR exon 20 C797S mutation was a major resistance mechanism to osimertinib.^[17]^ C797S may disrupt the key cysteine residue at the drug-binding site. Although fourth-generation EGFR-TKIs such as EAI045 have been developed for the C797S mutation,^[18]^ there are currently no available drugs against T790 M/C797S in the clinic.

PD-L1 high expression (80%) was detected after treatment with the first- and second-generation TKIs in this patient. Some studies have shown that PD-L1 high expression in tumors may predict a poor response to targeted therapy in EGFR-mutated NSCLCs.^[19,20]^ The role of immunotherapy as a second-line therapy in EGFR-mutated NSCLCs after EGFR-TKIs is still controversial, because there is no overall survival improvement over docetaxel.^[21]^ The recent results of the IMpower150 phase III trial indicated that patients in the EGFR-mutated subgroup could benefit from bevacizumab, a monoclonal antibody directed against vascular endothelial growth factor (VEGF), chemotherapy, and atezolizumab after EGFR-TKIs.^[22]^

## 4 Conclusion

Herein, our report represents a remarkably interesting case of the complexity and heterogeneity of EGFR mutations along the treatment course of different TKIs via NGS-based tumor tissue and liquid biopsy. The patient underwent gefitinib, afatinib, and osimertinib combined with crizotinib treatment, and developed different EGFR resistance mutations. In conclusion, although osimertinib combined with crizotinib showed dramatic tumor shrinkage both in primary tumor and bone metastasis in EGFR T790M-mutant NSCLC patients with MET amplification, more studies should be performed to confirm the clinical benefit in PFS and overall survival. Repeated NGS testing during treatment may provide insight into clonal evolution and help to select the optimal drugs to achieve better overall clinical outcomes.

## Author contributions

**Conceptualization:** Dapeng Li, Qi Gui, Kai Chen.

**Data curation:** Caihua Xu.

**Resources:** Meng Shen.

**Supervision:** Kai Chen.

**Writing – original draft:** Dapeng Li, Qi Gui.
